# A97 LARGE NON-PEDUNCULATED COLONIC POLYP (LNPCP) OUTCOMES REFERRED FOR ENDOSCOPIC RESECTION IN BRITISH COLUMBIA: A QUALITY ASSURANCE INITIATIVE

**DOI:** 10.1093/jcag/gwac036.097

**Published:** 2023-03-07

**Authors:** L Tam, D Akhtar, E Hill, S Jiang, A Ghuman, W Xiong, N Shahidi

**Affiliations:** 1 GI; 2 Medicine; 3 Surgery; 4 Pathology and Laboratory Medicine, UBC, Vancouver, Canada

## Abstract

**Background:**

Endoscopic resection techniques have become the primary treatment strategy for the vast majority of large (≥ 20mm) non-pedunculated colonic polyps (LNPCPs). Despite this, surgery is still commonly performed with evidence suggesting an increasing trend over time. There is limited Canadian data confirming the effectiveness and safety of an endoscopic management strategy for LNPCPs.

**Purpose:**

To investigate clinical outcomes of patients referred for endoscopic management of a LNPCP.

**Method:**

Retrospective single-centre analysis of patients referred to a single endoscopist for the management of LNPCPs within a tertiary referral practice. LNPCPs were further subdivided into non-complicated (NC-LNPCP) or complicated (C-LNPCP) defined as those involving the ileocecal valve, appendiceal orifice, circumferential or previously attempted. Performance outcomes were evaluated by the frequencies of technical success (removal of all polypoid tissue during index procedure) and need for colorectal surgery. Safety was evaluated by the frequencies of clinically significant intraprocedural bleeding (CSIPB), clinically significant post-endoscopic resection bleeding (CSPEB), intra-procedural perforation and delayed perforation. Recurrence (either endoscopic or histologic) was evaluated at first surveillance colonoscopy (SC1). Continuous variables were summarized using median (IQR). Categorical variables were summarized as frequencies (%). To test for association between categorical variables, the Pearson χ2 or the Fisher exact test were used, where appropriate. For continuous variables, the Mann-Whitney U test was used. A probability (p) value of <0.05 was considered statistically significant.

**Result(s):**

Between January 2021 to March 2022, 263 LNPCP were referred for endoscopic resection and 41 LNPCP were excluded (23 pedunculated, 14 optical evaluation suggestive of deeply invasive cancer, 4 other). 222 LNPCP (188 NC-LNPCP, 34 C-LNPCP) underwent endoscopic resection. Median size was 25mm (IQR 20-30mm) with the majority undergoing cold snare resection (115, 51.8%). Polyposis (Adenomatous or serrated) was present in 23 (12.6%) cases respectively. Technical success was 97.3%. Cancer was present in 5 (2%). Clinically significant bleeding (CSPEB) occurred in 2.7%, DMI IV in 1.8% and there were no delayed perforations. Recurrence occurred in 4 (3.5%) at SC1 and 11 (5%) required surgery due to technical failure, submucosal invasion on pathology and clinically significant bleeding.

**Image:**

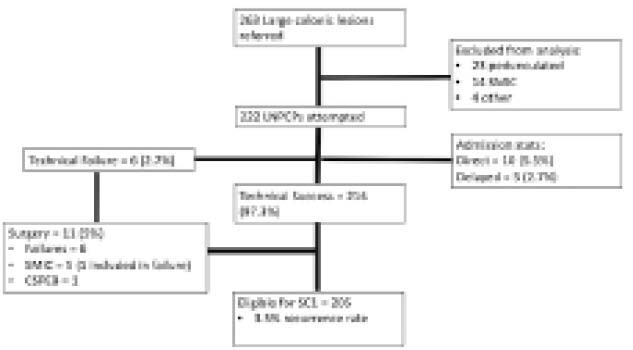

**Conclusion(s):**

Endoscopic resection as the primary treatment strategy for LNPCPs offers a safe and effective alternative to surgery in British Columbia.

**Please acknowledge all funding agencies by checking the applicable boxes below:**

None

**Disclosure of Interest:**

None Declared

